# Evaluating the Wear of Resin Teeth by Different Opposing Restorative Materials

**DOI:** 10.3390/ma12223684

**Published:** 2019-11-08

**Authors:** Seunglee Jin, Jae-Won Choi, Chang-Mo Jeong, Jung-Bo Huh, So-Hyoun Lee, Hyeonjong Lee, Mi-Jung Yun

**Affiliations:** Department of Prosthodontics, Dental Research Institute, Institute of Translational Dental Sciences, School of Dentistry, Pusan National University, Yangsan 50612, Korea; victoryjin528@gmail.com (S.J.); won9180@hanmail.net (J.-W.C.); cmjeong@pusan.ac.kr (C.-M.J.); neoplasia96@hanmail.net (J.-B.H.); romilove767@naver.com (S.-H.L.); hyunjongqqq@gmail.com (H.L.)

**Keywords:** dental restoration wear, tooth, artificial, gold alloys, zirconium oxide, lithia disilicate, nickel–chromium–beryllium alloy, feldspathic porcelain, steatite

## Abstract

The aim of this study was to evaluate the wear properties of resin teeth with different opposing dental restorative materials. One type of resin tooth (Trubyte Biotone) was tested against six types of restorative materials including type III gold alloy (GO), monolithic zirconia (MZ), lithium disilicate glass ceramic (LD), nickel–chromium alloy (NC), feldspathic ceramic (FC), and steatite (ST). Two-body wear tests were performed under a vertical load of 5 kgf and thermo-cycling at 5/55 °C with a total of 120,000 cycles. The wear amount was quantified by measuring the volume loss of the resin teeth and the vertical substance loss of the opposing materials using three-dimensional images. The FC group showed a significantly greater amount of wear of the resin teeth, followed by the ST, NC, LD, MZ, and GO groups. The GO group showed significantly less wear of resin teeth than the other groups. There were no statistically significant differences in the wear of opposing restorative materials between groups. Within the limits of this study, it is recommended that zirconia, rather than feldspathic ceramic, should be used for restorations in the esthetic zone, and gold alloy should be used for areas with little or no esthetic demand.

## 1. Introduction

Factors affecting the service life of a removable denture include the prognosis of the abutment teeth, discoloration, fracture of the resin teeth or denture base, and whether long-term maintenance is performed (e.g., periodic relining of the denture base) [1,2]. One of the most important and inevitable factors is the wear of the resin teeth. The wearing of resin teeth not only decreases the chewing efficiency but also the stability and retention of the denture. Resorption of the residual alveolar ridge occurs at a more rapid rate due to the uneven distribution of occlusal forces [3]. In addition, a decreased occlusal vertical dimension can lead to temporomandibular joint problems as well as accompanying changes in the facial profile [3,4,5,6]. In order to slow the rate of wear of resin teeth, materials with increasingly higher wear resistance have been developed [5,7,8]. Continual modifications to improve the low wear resistance of earlier developed acrylic resin teeth include the use of cross-linking agents, interpenetrating polymer networks with a three-dimensional network structure, and composite resin containing filler particles [9,10,11]. Nevertheless, the rate of wear of the resin teeth is also heavily dependent on a combination of other factors such as chewing, dietary, and parafunctional habits [12,13,14]. Furthermore, resin teeth incorporated into removable partial or complete dentures oppose either natural teeth or restored teeth. As wear is the phenomenon of the gradual removal of a material at solid surfaces that occurs as a result of relative motion occurring between two contact surfaces, the rate of wear of resin teeth will thus also depend on the various physical characteristics of the restorative materials, such as the strength, surface quality, and friction coefficient [15,16].

A number of previous studies have evaluated factors influencing the wear of resin teeth [10,11,17,18,19,20,21]. The majority of these studies, however, have investigated the wear resistance of either a single type or a range of resin tooth types against natural teeth or a single type of restorative material [10,11,17,18,19]. In contrast, there is a dearth of studies which have made direct comparisons of various restorative materials against resin teeth [20,21,22]. The aim of this study was to compare the wear of cross-linked polymethylmethacrylate (PMMA) based resin teeth when opposing a range of restorative materials commonly used in dentistry. The null hypothesis of this study was that there would be no significant differences in the amount of resin tooth wear between the different restorative materials tested.

## 2. Materials and Methods

### 2.1. Materials

Cross-linked PMMA based resin teeth, Trubyte Biotone (Dentsply Sirona, Philadelphia, PA, USA), were subjected to an antagonistic wear test involving six types of restorative materials: type III gold alloy (the GO group), monolithic zirconia (the MZ group), lithium disilicate glass ceramic (the LD group), nickel–chromium (Ni–Cr) alloy (the NC group), feldspathic ceramic (the FC group), and steatite ceramic (the ST control group). The product names and manufacturers are listed in Table 1.

### 2.2. Preparation of Specimens

#### 2.2.1. Fabrication of Specimens of Resin Teeth

**Figure 1:**
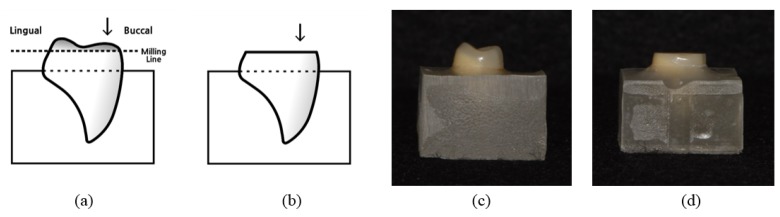
Schematic drawing of a resin tooth specimen. (**a**) The resin tooth was embedded in an orientation such that the occlusal surfaces of the mesiobuccal and distobuccal cusps were parallel to the horizontal (floor). (**b**) The embedded resin tooth was ground down evenly in height by 0.5 mm using a surveyor milling machine. Arrow indicates the direction of vertical load. (**c**) Embedded resin tooth with acrylic resin. (**d**) Prepared resin tooth specimen.

The first maxillary premolar resin tooth form was tested. A total of 48 specimens were fabricated, with eight resin teeth allocated per restorative material group. In order to minimize the amount of preparation on the buccal cusp (the area at which the wear test occurred), the resin teeth were embedded in an orientation such that the occlusal surfaces of the buccal cusps were parallel to the horizontal (floor) with an acrylic resin (Caulk Orthodontic resin, Dentsply Sirona, Philadelphia, PA, USA) using a uniform silicone (Express^TM^ STD, 3M Dental product, St. Paul, MN, USA) mold (15 mm × 15 mm × 10 mm) (Figure 1a,c). The embedded resin teeth were ground down evenly in height by 0.5 mm using a surveyor milling machine (Frasgerat F1, Degussa, Essen, Germany) under water to form a flat surface parallel to the floor (Figure 1b,d). The flat surface was polished with silicon carbide paper of #600 grit and #1000 grit under water cooling.

#### 2.2.2. Fabrication of Restorative Material Specimens

**Figure 2:**
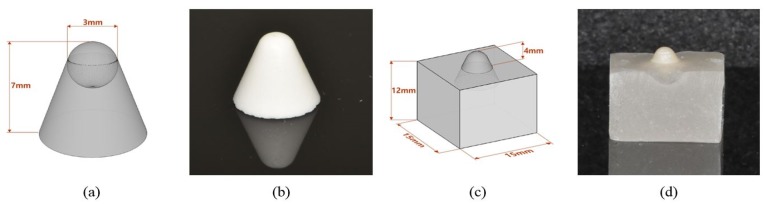
Schematic drawing of a restorative material specimen. (**a**) Standardized design for a restorative material specimen. (**b**) Steatite specimen fabricated by the CAD-CAM system. (**c**,**d**) Specimens embedded in auto-polymerizing acrylic resin.

In order to standardize the morphology of the opposing specimens, a conical shape was designed with a 3 mm diameter spherical tip using computer aided design (CAD) software (ThinkerCAD™, Autodesk, San Rafael, CA, USA) (Figure 2a,b).

For the GO group, wax specimens of the same design were fabricated with a computer aided design-computer aided manufacturing (CAD-CAM) system using wax blocks (MAZIX Wax, VERICOM, Gangwondo, Korea). The lost wax technique was then used to cast the wax specimens using type III dental gold alloy (Goldenian C-55, Sinhung, Seoul, Korea). Surface polishing was performed with a polishing kit (Gold polishing kit, Shofu, Kyoto, Japan).

The MZ group was fabricated in the same shape from zirconia blocks (Luxen, Dentalmax, Seoul, Korea) using a CAD-CAM system and subsequently prepared in a sintering furnace in accordance with manufacturer instructions (DuoTron Pro, B & D Dental Technologies, West Valley, UT, USA). Surface polishing was performed using a polishing kit (Porcelain Adjustment HP kit, Shofu, Kyoto, Japan).

For the LD group, heat pressable lithium disilicate glass ceramics (Amber press, HASS Co., Gangwondo, Korea) specimens were fabricated with the lost wax technique. Wax specimens of the same shape were prepared and invested. Then, lithium disilicate glass ceramic ingot was pressed at 900 °C in accordance with the manufacturer’s instructions. Prepared specimens were polished with a polishing kit (Porcelain Adjustment HP kit, Shofu, Kyoto, Japan).

The NC group was prepared by obtaining wax specimens in the same manner as for GO specimens and casting them with Ni–Cr alloys (Verabond 2V, Albadent, Fairfield, CA, USA). Surface polishing was carried out using a polishing kit (Gold polishing kit, Shofu, Kyoto, Japan).

For the FC group, Ni–Cr alloy specimens were first prepared as a cone shape with the tip of the sphere having a diameter 1 mm smaller than the previously described specimens. Porcelain (Ceramco, Dentsply Sirona, Philadelphia, PA, USA) was subsequently prepared by layering and firing in a conventional manner. The prepared specimens were subjected to surface polishing using a polishing kit (Porcelain Adjustment HP kit, Shofu, Kyoto, Japan).

The ST group was fabricated in the same design by the CAD-CAM system and subjected to surface polishing using a polishing instrument (Porcelain Adjustment HP kit, Shofu, Kyoto, Japan).

All fabricated specimens were embedded in semi-transparent acrylic resin (Caulk Orthodontic resin, Dentsply Sirona, Philadelphia, PA, USA) using a uniform silicone mold (15 mm × 15 mm × 10 mm) with the tip exposed to 4 mm (Figure 2c,d).

### 2.3. Methods

#### 2.3.1. Wear Test

**Figure 3:**
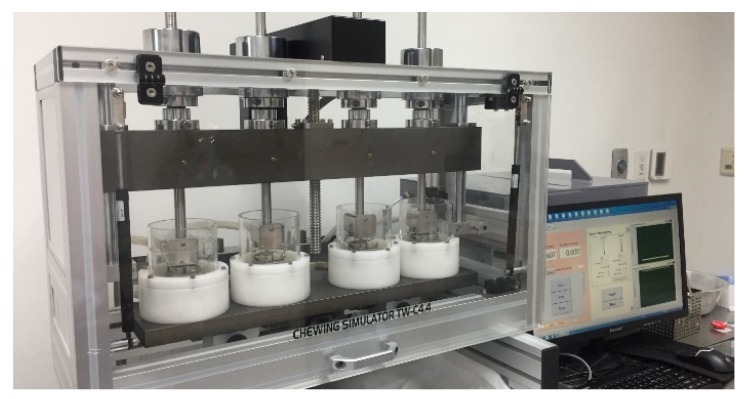
Dual-Axis Chewing Simulator (Chewing Simulator TW-C4.4, Tae-won Tech, Incheon, Korea)**.**

The wear test was conducted with a chewing simulator (Dual-Axis Chewing Simulator TW-C4.4, Tae-won Tech, Incheon, Korea) which repeatedly placed the resin teeth specimens into contact with the restorative material specimens using horizontal and vertical motions. Resin teeth specimens were fixed to the lower part of the chewing simulator. The restorative material specimens were fixed to the upper part, such that they could be placed into contact with the buccal half of the occlusal surfaces of the resin teeth. A standardized 0.7 mm horizontal was used to simulate natural masticatory motions. The vertical load was set at 5 kgf, and the reciprocating frequency was set at 1.2 Hz. Water was cycled by supplying it at intervals of 60 s at 5 °C and 55 °C. A total of 120,000 chewing cycles were completed for each set of resin teeth and restorative material specimens (Figure 3).

#### 2.3.2. Wear Measurement

**Figure 4:**
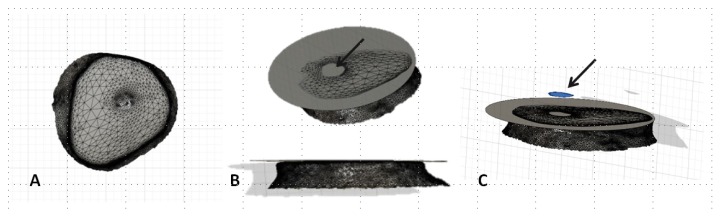
Resin teeth specimens were subjected to a surface scan using a three dimensional (3D) scanner for wear measurement. (**A**) A Standard Triangulated Language (STL) file of a resin tooth specimen after the wear test. (**B**) A new plane was defined by three points on a flat unworn surface. (**C**) The volume of space between the new plane and the worn surface was calculated. Arrow indicates the volume of loss.

After the wear test, the resin teeth specimens were subjected to a surface scan using a 3D scanner (TRIOS, 3Shape, Copenhagen, Denmark) in order to obtain a Standard Triangulated Language (STL) file. Using CAD software (Fusion 360, Autodesk, San Rafael, CA, USA) (0.001 mm^3^ accuracy), a new plane was defined by three points on a flat unworn surface, and the volume of space between the new plane and the worn surface was calculated (mm^3^) (Figure 4). The opposing restorative material specimens were scanned using a 3D scanner (TRIOS, 3Shape, Copenhagen, Denmark) both before and after the wear test. The two STL files were superimposed using a 3D program (3Shape 3D Viewer program, 3Shape, Copenhagen, Denmark) (1 μm accuracy), and the height difference between the two specimens at the cross section across the tip was obtained (mm).

#### 2.3.3. Scanning Electron Microscopy Evaluation (SEM) Analysis

The resin surface was observed after the abrasion test at magnifications of 50× and 1000× using SEM (Hitachi FE-SEM S-4700, Michigan Tech, Houghton, MI, USA).

### 2.4. Statistical Analysis

The wear amount of the resin and restorative materials were analyzed with a statistical analysis program (SPSS, IBM Corp., Chicago, IL, USA). The Shapiro–Wilk test and Levene’s test were performed to test the distribution normality of the data and variance homogeneities. A one-way ANOVA (α = 0.05) with Tukey’s post-hoc (α = 0.05) test was conducted to statistically test the differences between the groups. The level of statistical significance was set at *p* < 0.05.

## 3. Results

**Figure 5:**
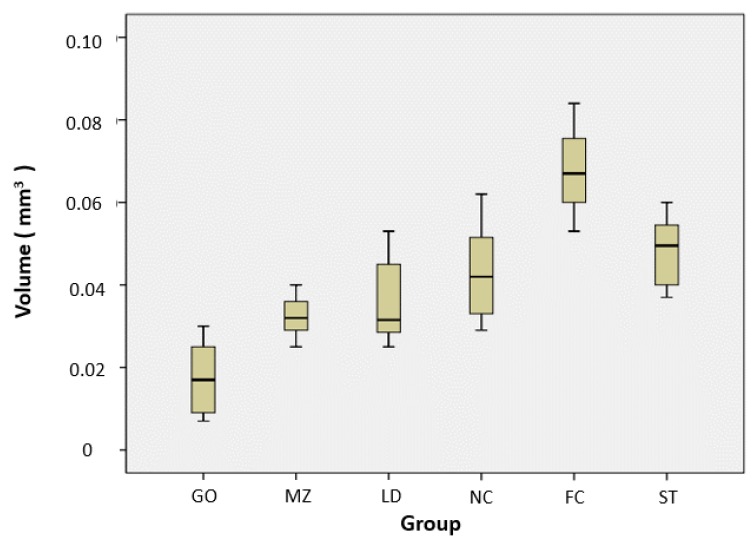
Volume loss distribution of resin teeth. GO, type III gold alloy group; MZ, monolithic zirconia group; LD, lithium disilicate glass ceramic group; NC, Ni–Cr alloy group; FC, feldspathic ceramic group; and ST, steatite group.

The means and standard deviations of the resin teeth wear measured in each group after the experiment are shown in Table 2. The wear of resin teeth in the FC group (0.068 ± 0.011 mm^3^) was the greatest and was significantly higher than that of the other groups. This was followed by the ST group (0.048 ± 0.009 mm^3^), the NC group (0.043 ± 0.012 mm^3^), the LD group (0.036 ± 0.010 mm^3^), the MZ group (0.032 ± 0.005 mm^3^), and the GO group (0.017 ± 0.009 mm^3^). The GO group showed significantly less wear of the resin teeth compared to the other groups. There was no significant difference between the lithium disilicate glass ceramic, Ni–Cr alloy, and steatite groups (Figure 5).

**Figure 6:**
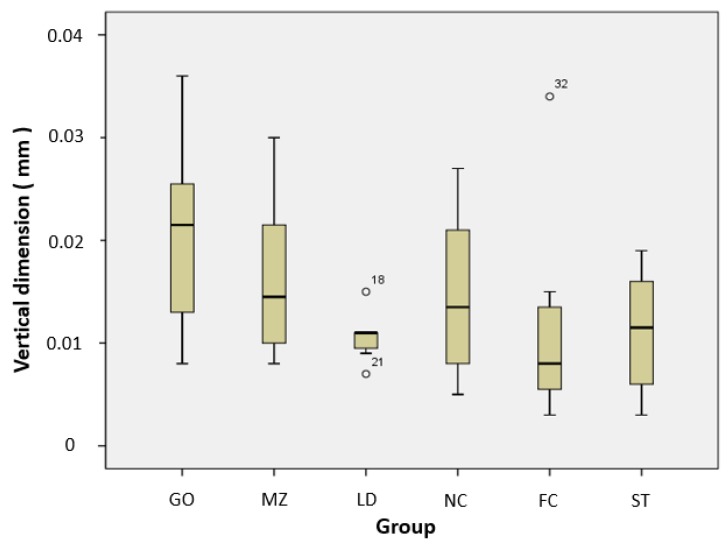
Vertical dimension loss distribution of restorative materials. GO, type III gold alloy group; MZ, monolithic zirconia group; LD, lithium disilicate glass ceramic group; NC, Ni–Cr alloy group; FC, feldspathic ceramic group; and ST, steatite group.

The average and standard deviation of the vertical dimension loss of the restorative material specimens after the wear test are shown in Table 2. There were no significant differences between groups (Figure 6).

**Figure 7:**
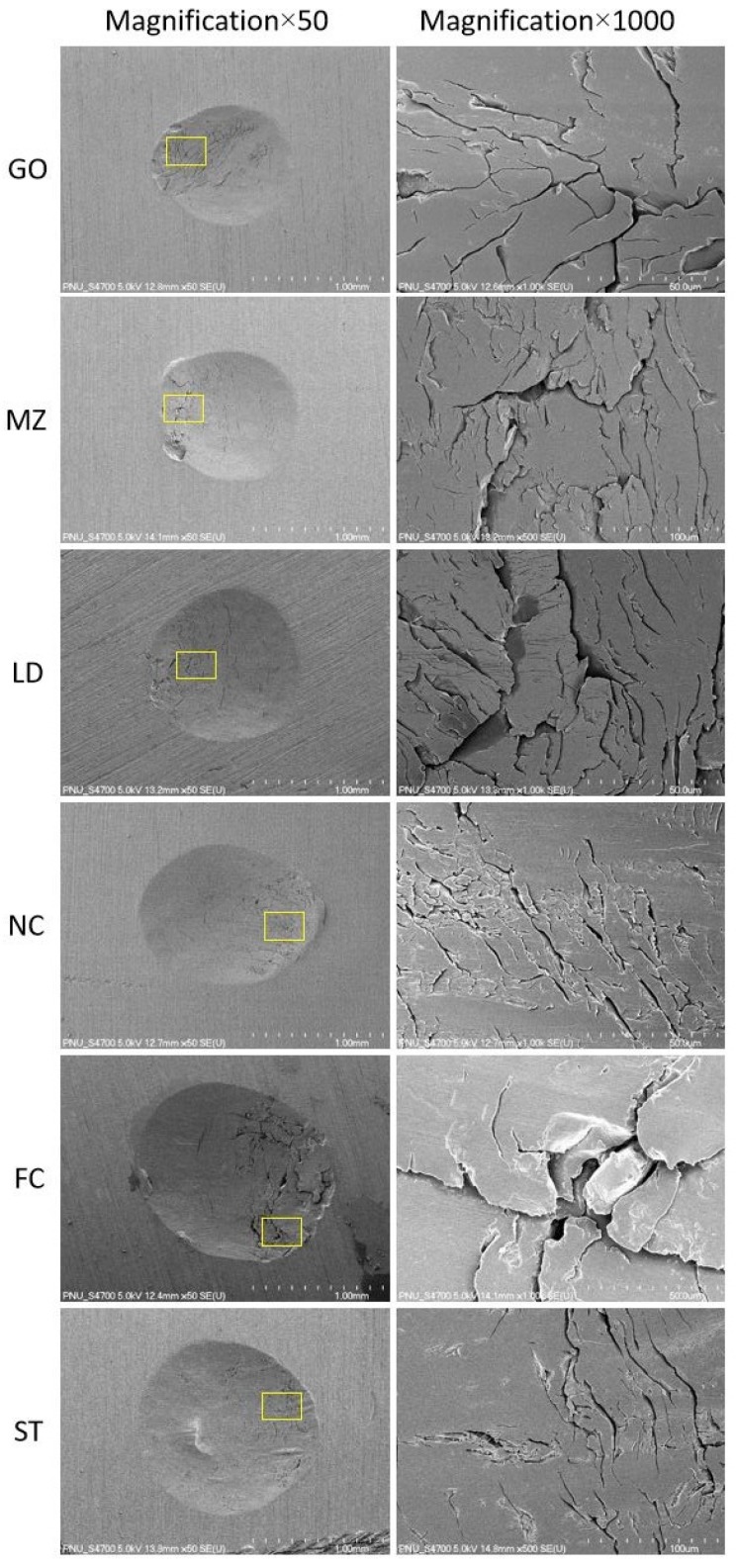
SEM images of the worn surface of the restorative materials. GO, type III gold alloy group; MZ, monolithic zirconia group; LD, lithium disilicate glass ceramic group; NC, Ni–Cr alloy group; FC, feldspathic ceramic group; and ST, steatite group.

SEM images of the resin surfaces after the wear tests are shown in Figure 7. Cracks were frequently observed among the restorative materials after vertical loading. Deep cracks and a greater degree of particle chipping were more commonly observed in the FC group compared to the other groups. In the majority of the groups, lateral movements were associated with a smooth surface.

## 4. Discussion

The purpose of this study was to compare and evaluate the wear of resin teeth when opposed by different restorative materials. In accordance with the results of this study, the null hypothesis of there being no significant differences in the amount of resin teeth wear between the different restorative material groups was rejected.

Evaluation of the wear of resin teeth may be performed through either in vitro or in vivo methods. While in vivo experiments may reproduce the oral environment where the actual resin wear occurs, standardization of the methodology and the generation of reliable results have proven to be difficult due to intra- and inter-subject variations in oral temperature, saliva composition, saliva pH, chewing habits, and dietary habits. Therefore, the use of an in vitro methodology in the current study allowed greater control of confounding variables. Two-body wear test methods were used to evaluate the amount of wear due to direct contact between the resin teeth and the different restorative materials [9]. The chewing simulator used in this experiment is a device capable of repeating vertical movements that simulate the mandibular closing movement as well as horizontal movements that simulate lateral excursion. The vertical load was set to 50 N, which is the average masticatory load of healthy individuals without abnormal function [23]. The number of cycles was set to 120,000, which accounted for approximately half of the average number of chewing cycles (250,000) generated by a single individual over one year [24]. During the wear test, the water was periodically alternated between 5 and 55 °C to simulate average fluctuations in the oral environment that would be expected over the course of a dental restoration’s service life, and residues generated by friction and wear were also regularly removed from the specimen surfaces.

A wide range of resin teeth with excellent wear resistance are currently commercially available and widely used in dentures [9,25]. In this study, we used Trubyte Biotone, a cross-linked acrylic resin tooth that is commonly used by clinicians due to its good physical properties and reasonable cost. In terms of the choice of restorative materials to be tested, an attempt was made to include as many of the most commonly used dental restorative materials (gold alloys, Ni–Cr alloys, feldspathic ceramics, lithium disilicate ceramics, and monolithic zirconia) as was feasible. While it was possible to use natural tooth enamel as the control, standardization would have been difficult as tooth shape and composition are not uniform. Instead, steatite, a material that has similar physical characteristics to enamel and can be used in place of enamel in abrasion tests, was used [11,26,27]. In contrast with natural enamel, the shape of the steatite specimens could be standardized and produced by the CAD-CAM system.

This study resulted in varying amounts of wear on the resin teeth depending on the type of restorative material tested. Of the six different restorative materials, feldspathic ceramic exhibited the greatest wear on resin, followed by steatite, Ni–Cr alloy, lithium disilicate glass ceramic, monolithic zirconia, and gold alloy. According to Oh et. al., the degree of wear is affected by the surface structure and surface roughness of the material [5]. Krejci et. al. also reported that wear is affected by the hardness, texture, and surface finish of the material [28]. Previous reports have reported that feldspathic ceramics cause a lot of wear on the opposing teeth due to the high hardness (Vickers hardness of 4.9 GPa), high surface roughness, and by-products resulting from wear [18,29]. According to Schuh et. al., high friction coefficients between porcelain and opposing surfaces may increase fatigue and wear [30]. Therefore, while feldspathic ceramics may have the advantages of high wear resistance and low fracture resistance, they are also associated with a high degree of wear of opposing teeth which is attributed to both its physical hardness and surface properties.

Zirconia has become a widely used restorative material because of its esthetic and translucent properties while maintaining adequate strength. These properties enable it to be used as single crown restorations in both esthetically demanding anterior areas as well as posterior regions [31]. Good physical and surface properties (Vickers hardness of 13.24 GPa, flexural strength of 800–1000 Mpa) of monolithic zirconia ensured the maintenance of a highly polished surface as the wear tests progressed [15,16,20]. As a result, the frictional coefficient between zirconia and resin teeth remains lower. These characteristics resulted in less wear than feldspathic ceramics and all other tested materials, with the exception of gold alloy. Therefore, zirconia is recommended as a restorative material for teeth located in the esthetic zone, especially in opposing resin teeth.

Gold alloys have traditionally been widely used as they cause the least wear on opposing natural teeth [29,32]. In this study, gold alloys were also found to cause the least wear on resin teeth. Unless there are high esthetic demands due to tooth location or patient preference, gold alloys can be the first choice as an extracoronal restorative material when opposing resin teeth. Steatite has been used as one of the abrader materials in several previous wear experiments [11,17,26,27]. Wassell et al. reported that steatite can be used in place of enamel in abrasion tests because of its similar Vickers hardness and coefficient of friction [26]. Nevertheless, among the six tested restorative materials, steatite caused the second highest amount of wear on resin teeth. This may have been due to differences in surface roughness attributed to the specimen fabrication process.

One of the limitations in this study is the use of the intraoral scanner for measuring the vertical loss of the restorative materials. Several studies have reported that the Trios 3D scanner used in this experiment has higher precision and accuracy than other scanners [33,34]. However, according to Muller et. al. [35], the accuracy of intraoral scanners can vary according to the scanning strategies. Gimenez et. al. [36] also found that the accuracy varies depending on the skill of the person performing the scan. Therefore, there is a limitation in the accuracy of scanning the surface of restorative materials before and after wear test and a comparison between the different restorative materials.

While the present study was an in vitro experiment that attempted to simulate in vivo oral conditions, other confounding variables not taken into consideration may have affected the results. In addition, if the experiment was conducted using a three-body wear method in which a third material was placed between the resin teeth and opposing restorative materials, the results may have been different. Furthermore, this study tested several restorative materials against only one type of resin tooth. Further research on additional newly developed restorative materials and resin teeth are needed to inform clinical treatment planning, with the aim of improving the service life of prosthodontic treatment.

## 5. Conclusions

Within the limits of this study, the following conclusions can be drawn. Gold alloys cause the least wear on opposing resin teeth, while feldspathic ceramics cause the most wear. While there were no significant differences between zirconia, lithium disilicate and Ni–Cr alloys, zirconia showed less wear than lithium disilicate. Therefore, it is recommended that zirconia, rather than feldspathic ceramic, be used for restorations in the esthetic zone, and gold alloy be used for areas with little or no esthetic demand.

## Figures and Tables

**Table 1:** Materials examined in this study.

Materials	Group (Sample Size)	Vickers Hardness (GPa)	Product Name	Manufacturer
Resin teeth	-	-	Trubyte Biotone	Dentsply, Philadelphia, PA, USA
Type III gold alloy	GO (n = 8)	0.88–1.17	Goldnian C-55	Shinhung, Seoul, Korea
Monolithic zirconia	MZ (n = 8)	13.24	Luxen	Dentalmax, Seoul, Korea
Lithium disilicate	LD (n = 8)	5.8	Rosetta SM	HASS, Gangwondo, Korea
Ni–Cr alloy	NC (n = 8)	3.7	Verabond 2V	Albadent, Fairfield, CA, USA
Feldspathic ceramic	FC (n = 8)	4.97	Ceramco	Dentsply, Philadelphia, PA, USA
Steatite	ST (n = 8)	5.8	-	Yamamoto trading, Seoul, Korea

**Table 2:** Mean values and standard deviations of volume losses (mm^3^) of resin teeth and vertical substance losses (unit: µm) of opposing restorative materials.

Mean ± SD			
Group	N	Volume Loss of Resin Teeth (mm^3^)	Vertical Loss of Restorative Materials (µm)
GO	8	0.017 ± 0.009 ^a^	0.016 ± 0.008 ^a^
MZ	8	0.032 ± 0.005 ^b^	0.015 ± 0.008 ^a^
LD	8	0.036 ± 0.010 ^b c^	0.011 ± 0.002 ^a^
NC	8	0.043 ± 0.012 ^b c^	0.015 ± 0.011 ^a^
FC	8	0.068 ± 0.011 ^d^	0.020 ± 0.009 ^a^
ST	8	0.048 ± 0.009 ^c^	0.011 ± 0.006 ^a^
*p*	-	<0.001	

Same superscript letters were not significantly different (*p* < 0.05). GO, type III gold alloy group; MZ, monolithic zirconia group; LD, lithium disilicate glass ceramic group; NC, Ni–Cr alloy group; FC, feldspathic ceramic group; ST, steatite group.
